# Self-care behaviors in older adults suffering from knee osteoarthritis: Application of theory of planned behavior

**DOI:** 10.3389/fpubh.2022.958614

**Published:** 2022-11-04

**Authors:** Hanieh Jormand, Nasim Mohammadi, Ali Khani Jeihooni, Pooyan Afzali Harsini

**Affiliations:** ^1^Autism Spectrum Disorders Research Center and Clinical Research Development Unit of Shahid Beheshti Hospital, Hamadan University of Medical Sciences, Hamadan, Iran; ^2^Department of Public Health, School of Health, Fasa University of Medical Sciences, Fasa, Iran; ^3^Nutrition Research Center, Department of Public Health, School of Health, Shiraz University of Medical Sciences, Shiraz, Iran; ^4^Department of Public Health, School of Health, Kermanshah University of Medical Sciences, Kermanshah, Iran

**Keywords:** knee osteoarthritis, self-care behaviors, theory of planned behavior, educational intervention, patients

## Abstract

**Background:**

Osteoarthritis is one of the main reasons causing disablement. Educational intervention for self-care behaviors of patients suffering from knee osteoarthritis is important because its effect on quality of patient life decreases the economic burden of disorder on society and family. This study aimed to investigate the effect of educational intervention based on the theory of planned behavior (TPB) on promoting self-care behaviors in elderly patients suffering from knee osteoarthritis.

**Methods:**

This quasi-experimental study was performed on 200 elderlies suffering from knee osteoarthritis in the rheumatology clinics of Shiraz, Iran, in 2019. The subjects were divided into two groups (100 experimental and 100 control). Before and after 4 months, both experimental and control groups filled a questionnaire. After administering a pre-test to both groups, only the experimental group was trained based on the TPB constructs on self-care behaviors in elderly people suffering from knee osteoarthritis in eight sessions by presenting educational films and images, power points, and group discussions for solving problems.

**Results:**

The mean age of the experimental group was 67.25 ± 3.64, and the mean age of the control group was 66.12 ± 3.50. The average scores of attitudes, subjective norms, perceived behavioral control, intention, and behavior before the educational intervention did not have significant differences in experimental and control groups, however, 4 months after the educational intervention, the paired *t*-test indicated significant enhancement in every construct in the experimental group, but no significant changes in the control group.

**Conclusion:**

According to the results, the educational intervention increased the self-care behaviors of patients suffering from knee osteoarthritis based on the theory of planned behavior. Therefore, the results of this study can be used in theory-based intervention strategies for self-care behaviors of patients suffering from knee osteoarthritis.

## Background

Knee osteoarthritis is a musculoskeletal condition that affects older adults and causes pain, physical disablement, and life quality reduction (1). Severe osteoarthritis is painful and it may result in disability, and even affect life expectancy (2). Population aging has been accompanied by a rise in non-communicable diseases including osteoarthritis (3). Almost two-thirds of the participants among 454 elderly recruited suffered from knee OA (292, 64.3%) (4).

Investigations have revealed that the prevalence of knee osteoarthritis among people aged 65 or more is 60–90%; however, women tend to need more healthcare, have higher osteoarthritis prevalence, and more pain and inflammation (5). In 2008, in a study performed on 10,291 people in Tehran, Iran, the prevalence of knee osteoarthritis was reported at 15.3% (6). Most patients suffer from pain, muscle stiffness, weakness, inability to climb and come down stairs, and reduced performance, which reduces the quality of the patient's life due to its chronic, painful, and weakening nature. Because pain and performance reduction blunt the walking speed of patients, the purpose of osteoarthritis treatment is to reduce the pain by improving performance and saving the mobility of joints. By modifying this issue, the improvement of the motion and walking velocity of the patient will increase (7–9). Osteoarthritis is one of the major causes of disablement. In the United States of America, almost 43 million people and 24% of all hospitals' clearances and 4,000 days of treatment in hospitals are related to osteoarthritis (10). Age, body weight, type of job, metabolic diseases, and trauma are significant factors causing knee osteoarthritis. This disease negatively affects walking, climbing stairs, and bearing weight disorders, limiting older people's mobility (10). Other symptoms include the reduction of general performance, especially in osteoarthritic organs, stiffness, mobility reduction, instability, and buckling of knees. Limitations in activity may cause limitations in social performance and problems in internal organs, such as the heart and lungs, various muscles, housekeeping, shopping, traveling, exercising, and working. Lack of exercise is the major cause of chronic disease (11).

In old age, osteoarthritis patients are mostly exposed to continual limitations in daily life activities (ADL) (12). Retaining the physical performance for performing self-care activities in patients suffering from chronic diseases such as osteoarthritis is essential; hence, regular exercise for improving physical power effectively reduces pain and symptoms and improves patients' mobility (13, 14). Spector et al. (15) and Szoeke et al. (16) revealed a relationship between physical activity and knee osteoarthritis, i.e., physical activity may cause the enhancement or reduction of knee osteoarthritis (17). Felson (18) and Hootmen et al. (19) stated that physical activity might not be efficient, as in studies among special populations of elite athletes as well as the general population show that long-term and vigorous physical activity and specific, strenuous sport participation increase the risk of developing hip and knee osteoarthritis. Rogers et al. (20) and White et al. (21) declared that physical activity might save knee joints from degenerative changes. Self-care is important in helping patients become independent, adapt to their disease, and improve their quality of life (22). Patients' behaviors, performances, and reactions are different in various conditions (23). Physical activity (24) and weight control are two key ways of managing knee osteoarthritis and hip muscles (22). However, those who will be able to administer self-care for their osteoarthritis take the responsibility and side-effects of the disease and successfully combine the self-management behaviors in osteoarthritis with their daily activities (25). The results of Hansson et al. (26) and Coleman et al. (27) indicated that the educational self-management program improves the performance of patients suffering from arthritis and osteoarthritis. To combat the negative effects of osteoarthritis on the physical, psychological, and daily life of older adults and the economic burden caused by this disease, there is a need to have more self-management programs and non-medical treatment (28). Educational intervention for self-care behaviors of patients suffering from knee osteoarthritis is important because its effect on the quality of patient life decreases the economic burden of disorder on society and family. The stress on self-care methods on dangerous factors must be modifiable (29). The specialists in health education believe that the notable point for changing and modifying lifestyle begins by changing the incorrect behaviors and following and continuing the healthy behaviors in the long-term (29). By changing lifestyle, reducing weight, and partaking in proper exercise, this disease's pain and symptoms will reduce (30). Therefore, by determining a proper and healthy lifestyle, this disease's negative effects and problems can be decreased (31).

It is important to select an appropriate model to achieve the educational aims (32). Using the most appropriate theory significantly increases the efficient application of health education and promotion. It helps the specialists understand the effective factors of healthy behaviors, find a good purpose for interventions, and develop strategies and educational content (33). One of the theories for educational intervention to bring about behavioral changes is the theory of planned behavior, by modifying the individuals' intentions. Based on this theory, the most important factor for determining the individual's behavior is his/her intention, which is influenced by three constrictions of attitude, subjective norms, and perceived behavioral control (32, 34). Ajzen, the proponent of this theory, believed changes in intention and attitude toward subject norms will shape an individual's behavior. Also, evidence proved that in addition to the intention, the perceived behavioral control is an important variable that affects self-care behaviors (35, 36). There are various strategies performed by the theory of planned behavior for knee osteoarthritis (37, 38). The self-care behavior is more important in vulnerable patients, such as older adults and women (39). Hence, the present study was designed based on the theory of planned behavior and educational intervention to promote self-care behaviors in older adults suffering from knee osteoarthritis.

## Methods

### Design and sample

The present research is a quasi-experimental investigation, and the intervention/experimental group comprised older adults aged above 60 with knee osteoarthritis in 2019. The sample size was fixed at 75 subjects each for the intervention group and the control group, with a confidence level of 95% and test power of 80% based on similar studies (25, 40), with a means for self-care behaviors of 49.53 and 57.17 and standard deviations of 7.36 and 8.83 for the intervention group before and after the intervention. To account for the attrition rate, 100 subjects were to be selected for each group.


(1) $$\begin{array}{l} n=\frac{{\left(Z_{1-\frac{\alpha }{2}}+Z_{1-\beta }\right)}^{2}\left(\delta_{1}^{2}+\delta_{2}^{2}\right)}{{\left(\mu_{1}-\mu_{2}\right)}^{2}} \end{array}$$

The studied samples are patients who were referred to the rheumatology clinics in Shiraz, Iran. One clinic was randomly selected as the experimental group and the other one as the control group. The inclusion criterion of the present study is patients suffering from elementary osteoarthritis older than 60 years of age who consent to participate in the survey, who live in Fars's province, who have no history of an intra-articular injection, and who have no history of mental illness. The exclusion criterion include participants who are absent in more than two educational sessions, lack of discrimination by specialists, and those living in other provinces.

### Study instrument and measures

This quasi-experimental study was performed on 200 elderly patients suffering from knee osteoarthritis in the rheumatology clinics of Shiraz, Iran, in 2019. The subjects were divided into two groups (100 experimental and 100 control). Before and after 4 months, both experimental and control groups filled out a questionnaire. After administering a pre-test to both groups, only the experimental group was trained based on the TPB constructs on self-care behaviors in elderlies suffering from knee osteoarthritis in eight sessions by presenting educational films, images, power points, and group discussions for solving problems. Participation in this study was voluntary, and subjects were ensured that their information would remain confidential. Notably, after selecting experimental and control groups, a questionnaire was filled by two groups, and then the educational intervention was performed on the experimental group. The educational program included eight sessions about taking self-care behaviors in knee osteoarthritis based on the theory of planned behavior. One educational session was held as an educational workshop by physiotherapists, and in one of the sessions, a family member was present. At the end of the sessions, an educational booklet was given to the experimental group. An educational message was sent to the subjects once a week. Four months after the educational intervention, experimental and control groups filled out a questionnaire. After this process, it is essential to mention all educational materials offered to control groups.

The tool used for gathering information was a questionnaire with two sections. The first section had eight questions about demographic information such as age, sex, educational level, marital status, BMI, job, family history in osteoarthritis, and disease duration.

The second section included questions for measuring the constructs of the theory of planned behavior, such as attitude, subjective norms, perceived behavioral control, intention, and behavior. The suggestion of Ajzen was used to design a questionnaire about the theory of planned behavior as the basis for making each construct in the research tool (41). The theory of planned behavior was used in two previous studies (37, 39). The questionnaire was filled out by self-reporting. The content validity of items was validated by a survey carried out by health specialists and rheumatologists (*n* = 12). The consistency of the questionnaire was confirmed by performing an elementary study on 30 patients suffering from knee osteoarthritis (out of participants of this study) through Chronbach's alpha. The alpha domain for studied constructs was from 0.77 to 0.88, indicating the tool's acceptable internal stability. Knowledge questions were 12 multiple-choice items; the correct answer had one score, and the incorrect answer had zero scores (ranging from 0 to 12). Fifteen items were about measuring the attitude based on a five-point Likert scale from 1 (completely disagree) to 5 (completely agree), ranging from 15 to 75 points. For measuring the subjective norms, 15 items were used. For example, “My friends believe that I should have proper weight for self-care in knee arthritis” or “My family believes that I have to use fewer stairs for self-care in knee arthritis.” This section was evaluated based on a Likert scale from 1 for “completely disagree” to 5 for “completely agree” (ranging from 15 to 75 points). Fifteen items were used for measuring perceived behavioral control. For example, “Because of time limitations, regular exercising is impossible for me.” These items were measured based on a Likert scale from 1 for “completely disagree” to 5 for “completely agree” (ranging from 15 to 75 points). For evaluating the intention, 10 items were used, such as “I decide to have regular exercising for self-care for knee osteoarthritis” (ranging from 10 to 50 points). The questionnaire about self-care behavior included 12 exclusive questions ranging from 1 to 5, from “never” to “always” (ranging from 12 to 60 points). The scores of all constructs were calculated in percentage.

### Developed educational intervention

In addition to the usual observations on the experimental group, eight educational sessions (45–50 min) were held once a week in the health center by presenting educational films and images and power points, and group discussions for solving problems. To prevent the older adults from getting tired, most of the material was presented in the form of videos, clips, and pictures, and the least in the form of lectures. In these sessions, patients were encountered with examples of daily life problems, and with the help of researchers and group discussions, proper solutions were presented. Hence, patients participated actively in finding the solutions. The researcher engaged the participants by asking and answering questions, talking about their positive and negative experiences, and discussing the potential problems caused by the disease. Therefore, participants learned about practical education, and their problems were solved with the help of other subjects. The previous issues were reviewed at the beginning of every session, and the patient's questions were answered. At the end of each session, an educational booklet including the educational contents was given to the subjects, and patients were asked to practice the presented educational content in their homes. In the end, the experimental group had 4 months to practice the educational content. The researcher contacted them by phone to ensure they were performing the educational issues. After 4 months, both experimental and control groups filled out a questionnaire by interviewing.

It should be noted that the control group only received the usual observations. For ethical considerations, the mentioned educational booklet was given to the control group at the end of the investigation. The presented educational program of the experimental group is illustrated in Table 1.

**Table 1:** Educational self-care program.

**Educational self-care program**	
Ways to reduce pain	Relaxing the knee, using a hot water compressor, holding up the knee, reducing hard activities, modifying lifestyles such as sitting on a chair, using a cane, lying on a medical mattress, etc.
Educating appropriate diet	Use fresh fruits and vegetables, vitamins C and D, calcium, omega 3, soya, olive oil, drink enough water, green tea, etc.
Educating exercises	Walking, physical activities for improving knee joints (these activities were performed in every session by patients in the presence of the researcher)

One of the barriers to educational interventions for osteoarthritis patients was non-adherence to educational interventions (42). To overcome this barrier, the educators focused on the quality of the relationship between patients and doctors, and holding the educational interventions in the rheumatology clinics, with equal emphasis on treatment and education.

### Data analysis

The obtained results were analyzed by SPSS 22 software. To analyze the data by using descriptive statistics (the average and deviation criteria), an independent *t*-test, Chi-square, and paired *t*-test with a significant level of (*p* < 0.05) were used.

## Results

First of all, the normality of data checked Kolmogorov–Smirnov test showed that data were normally distributed (43, 44). The obtained results indicate that the average age of the experimental group is 67.25 ± 3.64 and the average age of the control group is 66.12 ± 3.50 (*p* = 0.189). The average BMI of the experimental group is 26.52 ± 3.35, the control group is 26.04 ± 3.28 (*p* = 0.292), and the independent *t*-test does not show any significant differences between the two groups. According to the Chi-square test, the two groups do not have significant differences in educational level (*p* = 0.315), marital status (*p* = 0.309), and sex (*p* = 0.187). The demographic characteristics of participants are presented in Table 2.

**Table 2:** Frequency distribution of studied subjects based on their personal information.

**Variables**		**Experimental group**		**Control group**		***P*-value**
		**Number**	**Percentage**	**Number**	**Percentage**	
Marital status	Single	2	2	4	4	*P* = 0.309
	Married	93	93	91	91	
	Divorced	3	3	3	3	
	Widow	2	2	2	2	
Educational level	Illiterate	5	5	3	3	*P* = 0.315
	Elementary	10	10	12	12	
	Guidance school	35	35	38	38	
	Diploma	36	36	34	34	
	Academic	14	14	13	13	
Job	Employed	31	31	35	35	*P* = 0.208
	Unemployed	69	69	65	65	
Disease duration	Under 5 years	35	35	39	39	*P* = 0.155
	Five years and more	65	65	61	61	
Family history in osteoarthritis	Yes	54	54	49	49	*P* = 0.090
	No	46	46	51	51	
Sex	Male	32	32	36	36	*P* = 0.187
	Female	68	68	64	64	

According to the independent *t*-test, the average scores of attitudes, subjective norms, perceived behavioral control, intention, and behavior before the educational intervention do not have significant differences in experimental and control groups; however, 4 months after the educational intervention, the paired *t*-test indicated significant enhancement in every construct in the experimental group. There remained no significant changes in the control group (Table 3).

**Table 3:** Comparing the average scores of knowledge, attitude, subjective norms, perceived behavioral control, intention, and self-care behaviors in experimental and control groups 4 months after the educational intervention.

**Variables**	**Scores**	**Group**	**Before intervention**	**Four months after the intervention**	***P*-value**
Awareness	(0–12)	Experimental	28.14 ± 5.36	68.12 ± 5.25	*P* < 0.001
		Control	29.5 ± 75.12	30.5 ± 14.26	*P* = 0.152
		*P*-value	0.216	*P* < 0.001	
Attitude	(15–75)	Experimental	35.4 ± 28.24	67.4 ± 34.85	*P* < 0.001
		Control	36.4 ± 51.28	72.4 ± 12.62	*P* = 0.258
		*P*-value	0.245	*P* < 0.001	
Subjective norms	(15–75)	Experimental	28.4 ± 32.28	72.4 ± 12.62	*P* < 0.001
		Control	27.4 ± 65.48	28.4 ± 36	
		*P*-value	0.275	*P* < 0.001	
Perceived behavioral control	(15–75)	Experimental	24.4 ± 33.16	68.4 ± 44.73	*P* < 0.001
		Control	24.4 ± 87.54	25.4 ± 91.60	*P* = 0.214
		*P*-value	0.322	*P* < 0.001	
Intention	(10–50)	Experimental	32.4 ± 28.54	69.4 ± 45.82	*P* < 0.001
		Control	33.4 ± 65.27	34.4 ± 65.48	*P* = 0.264
		*P*-value	0.381	*P* < 0.001	
Self-care behaviors	(12–60)	Experimental	25.4 ± 23.75	75.4 ± 12.82	*P* < 0.001
		Control	26.4 ± 15.96	27.4 ± 95.84	*P* = 0.214
		*P*-value	0.145	*P* < 0.001	

## Discussion

Population aging is accompanied by a rise in chronic diseases, including knee osteoarthritis, which causes pain and disability. The need for an efficient self-care program for these patients is becoming ever more pressing (45). This study aims to investigate the effect of educational intervention based on the theory of planned behavior on the promotion of self-care behaviors of older adults with knee osteoarthritis in Shiraz, Iran. They gave subjects information about methods for reducing pain, maintaining a proper diet, and exercising in order to increase the experimental group's knowledge. The results show that the average score of older adults' knowledge about self-care behaviors after the educational intervention was more significant in the experimental group than in the control group.

In the study of Taal et al. (46), group education caused an increase in knowledge and self-care behaviors of patients with rheumatoid arthritis. In the study of Saraboon et al. (47) on overweight patients suffering from knee osteoarthritis, the educational intervention caused significant promotion in their knowledge, healthy behaviors, reduction of knee pain, and weight of the experimental group. In the study of Hopmen-Rock and Westhoff (48) on patients with knee osteoarthritis, the educational intervention and exercise programs caused the enhancement of life quality and knowledge of the experimental group. In the study of Jeihooni et al. (49) on women older than 40 in Shiraz, the knowledge of subjects about preventive behaviors for knee osteoarthritis was less. Chen et al. (50) investigated the relationship between osteoarthritis patients' self-care behaviors and their knowledge. They concluded that there is no significant relationship between self-care behaviors and patients' knowledge. Notably, knowledge is an essential factor for behavior change but it cannot be a sufficient condition for behavior change alone (51). So, attention to other important factors to changing behavior, especially self-care behaviors, is essential.

Group discussion and presenting positive and negative experiences by subjects encouraged them to use self-care behaviors through modeling. To improve attitude, an educational picture booklet was given to the subjects and motivational messages were sent to them once a week. The results of this study show that, 4 months after the educational intervention, compared to the control group, the average score of the experimental group's attitude had significant enhancement. In the study of Mohammadizeidi et al. (52) on 150 computer users, the average score of attitudes of the experimental group had more significant enhancement than the control group. Morowatisharifabad et al. (53) investigated the attitude and self-care behaviors of patients suffering from knee osteoarthritis who were referred to the rheumatology clinics of Yazd, Iran. He found that the attitude of studied subjects was at an average level, and there was a positive harmony between the attitude and self-care behaviors of patients. So, another important factor in changing behavior is attitude (54); improving this variable through health education and promotion intervention programs and strategies was suggested.

In a review study by Kanvaki et al. (55), who investigated the barriers to facilitators of physical activities of patients suffering from knee osteoarthritis, positive attitude and positive exercising experiences, beliefs, and social supports were introduced as facilitators. The studies of Reddy et al. (56), Kwan et al. (57), and Kashfi et al. (58, 59) indicated the enhancement of the average score of subjects' attitudes after educational intervention. However, the study of Mazloumi et al. (39) showed educational intervention did not affect subjects' attitudes.

Based on the results from the present investigation, compared to the control group, 4 months after the educational intervention, the experimental group's average score of subjective norms was significantly enhanced. In this research, the educational sessions were held in the presence of rheumatologists and orthopedic specialists, and a family member was the effective person for performing self-care behaviors. Researchers carried out phone tracking with patients. By providing appropriate conditions and comprehensive support, families can help patients to employ self-care behaviors. Ethgen et al. (60) revealed a significant relationship between social support and life quality in patients suffering from knee osteoarthritis. In the study of Morowatisharifabad et al. (53), clinicians greatly affected the participation of patients suffering from knee osteoarthritis in exercising. The results of Morowatisharifabad et al. (61) showed no relationship between elderlies' self-care behaviors and subjective norms. In some studies, the educational intervention based on the theory of planned behavior causes an increase in the average score of subjective norms after educational intervention (62–65). However, in studies by Mazloumi et al. (39), Mohammadizeidi et al. (52), and Ali Mehri et al. (66), the educational intervention based on the theory of planned behavior does not affect the average score of subjective norms. Also, based on evidence, attitude, subjective norms, and perceived behavioral control have been able to predict 16.2% of the variance of self-care behaviors concerning knee osteoarthritis (61). Hence, designing educational programs to increase subjective norms among these patients is recommended.

In this research, 4 months after the educational intervention, the experimental group's average score of perceived behavioral control showed more significant enhancement than the control group. Perceived behavioral control includes an individual's confidence about his/her abilities for organizing activities and successful performance of considered behavior for obtaining a specific result in specific conditions. In other words, it indicates to what extent the individual's feeling about doing or not doing a specific behavior is under the control of him/herself. Whenever there is a limitation on performing a behavior, and the individual believes that he/she does not have sufficient or demanded facilities for doing that behavior, it is possible that, even by having sufficient knowledge, attitude, and subjective norms, he/she does not have a strong intention for doing that behavior (67). In other words, perceived behavioral control is the strongest predictor of knee arthritis self-care behavior (61). Therefore, improving strategies for providing educational programs among these patients might be effective.

Teaching new cognitive and practical skills through educational sessions and presenting encouraging feedback and proper information in group discussions caused the improvement of perceived behavioral control of the experimental group. In the quasi-experimental study of Mazloumi et al. (39) on female teachers by using the theory of planned behavior about preventive behaviors from knee osteoarthritis, after the educational intervention, the average score of perceived behavioral control was enhanced. Paruch (68) revealed a relationship between perceived behavioral control and intention of doing exercises in African-American overweight women. Gerayllo et al. (67) indicated a direct and significant relationship between perceived behavioral control and self-care behaviors of patients suffering from knee osteoarthritis. Robertson et al. (69) showed that agronomic education in the work environment significantly improved perceived behavioral control and body shape. In the study of Mohammadizeidi et al. (52), compared with the control group, the experimental group had higher scores in perceived behavioral control 3 and 6 months after the educational intervention. Williams et al. (70) investigated the effect of educational intervention based on the theory of planned behavior on the promotion of walking in subjects. They revealed that this intervention does not affect the subjects' perceived behavioral control, intention, attitude, and behavior.

Results of the present research indicated that, 4 months after the educational intervention, the average score of the intention of the experimental group for taking self-care behaviors had been enhanced. According to the theory of planned behavior, the increase in the average score of attitude, subjective norms, and perceived behavioral control in the experimental group 4 months after the educational intervention caused an increase in their intention, indicating the positive effects of educational programs. In studies by Mihamadizeidi et al. (52) and Allahverdipour et al. (71), the educational intervention based on the theory of planned behavior caused an increase in the behavioral intention of subjects. Mazloumi et al. (39) stated that the constructs of the theory of planned behavior predicted 37% of intention and 43% of preventive behaviors from knee osteoarthritis.

The current study results show that educational intervention based on the theory of planned behavior causes the promotion of self-care behaviors in elderly people suffering from knee osteoarthritis. The theoretical and educational program, by stressing on the effective factors for the promotion of self-care behaviors such as proper diet (using fruits and vegetables and foods including calcium), exercising (such as walking and improving knee joint), and using methods for reducing pain (relaxing the knee, holding up the knee, using a cane, etc.), along with group discussions and active participation of subjects, expressing benefits and barriers of self-care behaviors, increasing the practical skills and improving communicational skills, improving decision-making, solving problems, and learning appropriate behaviors with appropriate feedback improved the self-care behaviors of the experimental group.

Mirzaee et al. (40) investigated the effect of educational self-management programs on elderlies suffering from knee osteoarthritis. In his study, the experimental group received an educational session once a week for 4 weeks (70 min). After education, the experimental group had 4 months to practice the educational program in their homes. His results revealed that the experimental group's performance after the intervention was significantly more than the control group. In the study of Thomas et al. (72) on 786 men and women older than 45 years of age, the educational program caused the reduction of knee pain in studied subjects. In studies by Albaladejo et al. (73), Coleman et al. (74), French et al. (75), Jobnson et al. (76), Kroon et al. (77), Tak et al. (78), Tavafian et al. (79), and Nuñez et al. (80), the educational intervention caused the improvement of subjects' performance. In the study of Moazzami and Mohamadi (81), subjects' pain had significantly reduced by applying resistive changes in experimental group's variables. The study of Allegrante et al. (82) showed that the walking training program caused an improvement in the performance of patients who suffer from knee osteoarthritis.

Rocha et al. (83) investigated the effect of a selected practical program on pain, motion range, and the performance of patients suffering from knee osteoarthritis. He indicated a significant difference between the averages of pain, motion range, and knee joint performance before and after the practical program. Therefore, after the educational sessions, subjects' pain reduced, their motion range and performance improved. Toomey et al. (84) showed that group education by physiotherapists and exercise interventions cause the enhancement of self-care behaviors in patients with arthritis and chronic back pain. In a quasi-experimental study by Saffari et al. (37) on 120 patients with knee osteoarthritis, the educational intervention based on the theory of planned behavior caused the promotion of life quality of the experimental group 3 months after the educational intervention. In the study of Mazloumi et al. (39), the educational intervention based on the theory of planned behavior caused an increase in the average score of experimental group behavior. However, there seemed to be no significant changes in the control group.

## Conclusion

According to the results of this study, education based on the theory of planned behavior causes the promotion of self-care behaviors in older adults suffering from knee osteoarthritis. One of the advantages of this study is employing a community approach in performing self-care programs. Performing the presented interventions at home makes the suggested self-care programs available, acceptable, and useful for patients with low incomes. Also, this intervention allows an active role of patients in the self-care process.

One of the limitations of the current study was the subjects' self-reporting about their self-care behaviors. The other limitation was individuals' differences in answering the questions about the care and psychological support they received, which was out of the control of researchers. Also, living conditions significantly affect the performance of interventional programs at home, so harmonizing these performances was impossible for the researchers. Increased follow-up periods for future study in this field and sample selection in multi-centers instead of specialized rheumatology clinics are also suggested. In addition, comparison and analysis of country differences in health interventions and examining disability levels in educational and control groups of different studies for future study are suggested.

## Data availability statement

The original contributions presented in the study are included in the article/supplementary material, further inquiries can be directed to the corresponding author.

## Ethics statement

The studies involving human participants were reviewed and approved by Ethics Committee of Fasa University of Medical Sciences. The patients/participants provided their written informed consent to participate in this study.

## Author contributions

AK, HJ, NM, and PA assisted in conceptualization and design of the study, oversaw data collection, conducted data analysis, drafted the manuscript, and reviewed the manuscript. AK, HJ, and NM conceptualized and designed the study, assisted in data analysis, and reviewed the manuscript. All authors read and approved the final manuscript.

## Conflict of interest

The authors declare that the research was conducted in the absence of any commercial or financial relationships that could be construed as a potential conflict of interest.

## Publisher's note

All claims expressed in this article are solely those of the authors and do not necessarily represent those of their affiliated organizations, or those of the publisher, the editors and the reviewers. Any product that may be evaluated in this article, or claim that may be made by its manufacturer, is not guaranteed or endorsed by the publisher.

## Abbreviations

TPB: Theory of Planned Behavior
ADL: Activities of Daily Life.
